# Palliative Care and Multi-Agent Systems: A Necessary Paradigm Shift

**DOI:** 10.3390/clinpract13020046

**Published:** 2023-04-03

**Authors:** Kimberley C. Brondeel, Sheina A. Duncan, Patrick M. Luther, Alexandra Anderson, Pranav Bhargava, Chizoba Mosieri, Shahab Ahmadzadeh, Sahar Shekoohi, Elyse M. Cornett, Charles J. Fox, Alan D. Kaye

**Affiliations:** 1 School of Medicine, University of Texas Medical Branch, 301 University Blvd, Galveston, TX 77555, USA; 2 School of Medicine, Louisiana State University Health Sciences Center at Shreveport, Shreveport, LA 71103, USA; 3 Department of Anesthesiology, Louisiana State University Health Sciences Center at Shreveport, Shreveport, LA 71103, USA

**Keywords:** palliative care, chronic disease, multi-agent systems

## Abstract

Palliative care is intended to relieve caregivers of physical, psychological, and even spiritual elements of care. One of the most prevalent issues facing this form of care is a lack of healthcare resources and structures to deal with an aging population. This aging population is placing a strain on the healthcare system, prompting a need for a shift in system management. A potential answer to this issue may be the Multi-Agent System (MAS). This category of computerized networking system was created by programmers to gather relevant health information on a patient and allow for the system to act with other agents to decide the best course for disease management. It can also allow for a multidisciplinary healthcare team to make more informed plans of actions for their patients by providing accurate and up-to-date information resulting from a greater synergetic mesh. MASs could fulfill the demands of a rising chronic illness population and deliver high-quality care, indicating a major paradigm shift within the US. In this review, we will evaluate the aging population and contributing factors, palliative care and the need for the multi-agent system, and clinical considerations involving examples from healthcare systems both on and beyond US shores.

## 1. Introduction

**Figure 1:**
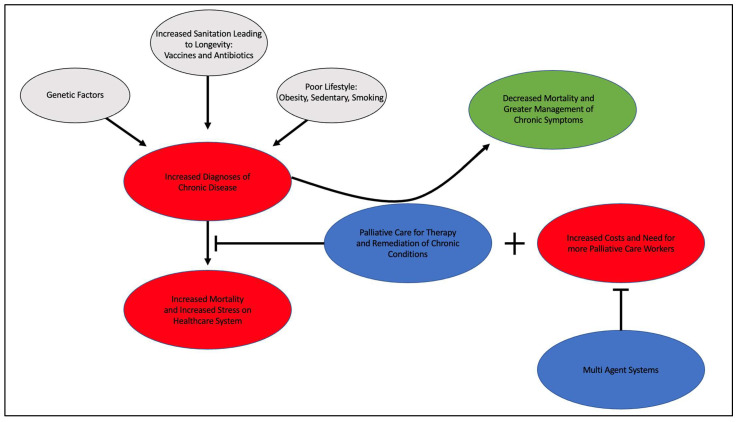
The increased prevalence of chronic disease from factors that contribute to greater longevity via vaccination and antibiotics, poor lifestyle, and genetic factors leads to increased mortality and stress on the healthcare system. The use of palliative care to treat and remediate chronic disease can lead to decreased mortality and greater management of symptoms than the current system can account for. Unfortunately, the increased volume of patients through palliative care would increase overall costs and need for more palliative care professionals; however, the use of multi-agent systems can mitigate these costs by providing the changes necessary to correct health course without need for human intervention and by providing a level of synergy to the healthcare system that is currently lacking.

Palliative care is a healthcare specialty developed in the 1960s to provide quality care and comfort to patients with serious and terminal diagnoses and has since become a staple for healthcare across the world [1]. It is designed to ease the burden of care on family members or friends, by providing for physical, psychological, and even spiritual aspects of care [1]. It can accompany the healthcare provided to those with diagnoses such as kidney disease, HIV, and cancer. A team consisting of physicians, nurses, mental health experts, and clergy or other religious professionals work together to address the patient’s needs [1]. With increased quality of life and greater symptom management, the usefulness of palliative care is widely recognized [2]. The US suffers from a chronic illness epidemic that may be remediated in part through expanded palliative care to those who suffer from it [3]. This expansion of palliative care will no doubt incur costs and require the need for increased palliative care workers. The Multi-Agent System (MAS) is designed to meet the needs of an expanding population of individuals with chronic illness and provide high-quality care by utilizing various agents to monitor the health of patients and make decisions independent of human intervention [4]. This model is designed to compensate for the increased resource cost, and thus could mitigate those with expansive palliative care. In planning to improve and expand palliative care in the US and worldwide, this model and other clinical considerations must be evaluated. The relationship of these various factors is displayed below (Figure 1). The prospect of an ever-increasing aging population that is plagued by chronic illness, the need for a shift in paradigm in the treatment of chronic illness via palliative care powered by a multi-agent system (MAS) of healthcare, and the benefits of a more holistic range of care are all factors to consider for system-wide changes of practice as we approach future decades. 

## 2. Chronic Disease and Contributing Factors

The prevalence of chronic disease within the United States has seen marked increases in the last few decades. In 2007, nearly half the population suffered from at least one chronic disease [5]. Data from 2022 suggests this number now encompasses almost 60% of Americans [6]. The staggering growth of an almost 10% increase in only 15 years’ time is related to multiple factors that have harmonized to produce a cacophonous dilemma, which is to say that the United States is facing a chronic disease epidemic. The contributing factors to this epidemic include increasing longevity due to improved sanitation/vaccines/antibiotics, poor lifestyle choices in the form of overeating/sedentation/smoking, and genetic factors that predispose one to chronic disease. 

The life expectancy at birth in 1900 was 51.5 and 58.3 for males and females, respectively [7]. The life expectancy at birth in 2021 was 73.5 and 79.3 for males and females, respectively [8]. Improvements in sanitation, nutrition, medications, and many other areas of public health have resulted in these increases [9]. Vaccinations have played a major role in increasing life expectancy in the last one hundred years. The protection conferred to those vaccinated is a critical resource in preventing infectious diseases before it can produce symptoms. This protection extends to individuals who have not been immunized and those who are immune-deficient and cannot receive vaccinations [10]. While therapeutics/vaccinations have resulted in marked increases in life expectancy, they have also unveiled a concern that eluded the forefront of minds throughout a significant portion of history: the potentiation of chronic disease. 

Another contributing factor to increasing chronic disease is the poor lifestyle seen within the US. Some of the high-risk factors for chronic disease that the CDC recommends avoiding are smoking, drinking, and a sedentary lifestyle [11]. Smoking habits within the US have seen decreases, from 20.9% in 2005 to 12.5% in 2020 [12]. The incidence of drinking within the US is nearly 50% with binge drinking at nearly 17% [13]. The prevalence of obesity in the US from 2017–2020 was 41.9%, with the prevalence in non-Hispanic black adults as high as 49.9% [14]. 

Genetic factors also contribute significantly to chronic disease within the US. In 2018, Wehby et al. studied the single-nucleotide polymorphism risk factors associated with chronic diseases such as coronary artery diseases, type 2 diabetes (T2D), obesity, rheumatoid arthritis, Alzheimer’s disease, and major depressive disorders using polygenic risk scores, and found significance in all groups except for the T2D group [15]. 

As the US struggles to treat these individual factors, the incidence of chronic disease is ever increasing. This places high demand on the healthcare system in the form of reactive rather than proactive care. When untreated, chronic illnesses progress gradually and thus individuals with chronic conditions require more long-term treatment. One such long-term treatment is palliative care, which is a branch of medicine that involves multidisciplinary care for the symptoms associated with advanced chronic illnesses. In the context of the US, palliative care is high-level care from a multidisciplinary team with efforts focused on curative ends [16]. Hospice care shares the same aspects as palliative care; however, it differs fundamentally in terms of comfort measures only because curative efforts have been largely abandoned [16]. Palliative and hospice care have been proven to improve the quality of life and medical care in end-of-life treatment [17]. With improved life expectancy and the contributing factors therein, the question of how these patients suffering from chronic illness will be cared for is of increasing import. The current medical system allots over 80% of its expenses to chronic disease treatment per year [18]. Despite the magnitude of the expenditures devoted to the treatment of chronic illness, data projects that the prevalence of chronic illness will continue to increase [5]. Therefore, the need for a new medical system, or one bolstered with a more powerful synergistic factor, is of great necessity in order to combat this growing epidemic. A potential candidate for this position may be the Multi-Agent System (MAS). With MASs, a system of agents can make predictions on the steps needed to improve the patient’s health and can be updated in real time at a central location, and thus may allow for an additional layer of synergism to be added to the healthcare schema [4]. The use of this system could greatly expand and make efficient the resources that are already present, mitigating the increased costs of instituting palliative care as a healthcare standard in the treatment of chronic disease.

## 3. Multi-Agent Systems and Palliative Care

The precise machinations in which a multi-agent system operates under and interacts are a topic all its own and is outside the scope of this review. There is a comprehensive article of great depth on the topic titled “A Roadmap of Agent Research and Development” by N. Jennings et al. 

**Figure 2:**
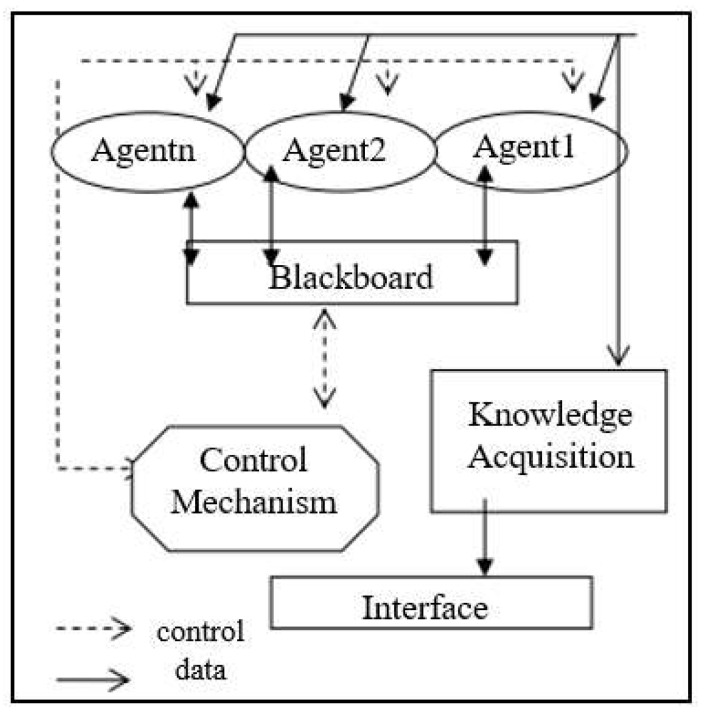
The blackboard model of Multi-Agent Systems. Data are able to flow between the Blackboard, Agent, and via request through the interface for knowledge acquisition. Control of the system occurs through the control mechanism, which can influence the blackboard and agents. Credit to Okba Kazar, “Multi-agents system for medical diagnosis” Reprinted/adapted with permission from Ref. [21]. 2018, Okba Kazar.

Multi-agent systems are computerized networks that use agents to cross-reference and monitor relevant health data on a patient and then proactively or reactively make changes to benefit the patient without the need for human intervention [4]. The “agents” referred to in this framework are individual programs that can use artificial intelligence techniques in order to act on and correct course to a desired goal or range, as programmed [4]. These agents are highly variable in their specified capability as interpreted by their given programming; however, all agents must have three aspects: sociability, pro-activeness, and responsiveness [19]. Sociability refers to the interaction of the agent with other agents, physicians, and patients in an effort to solve a problem. Pro-activeness is the ability to predict the need for action before prompted and make efforts to mitigate that eventuality. Responsiveness refers to the ability to perceive the directed environment and make changes [19]. The principal advantage and usefulness of multi-agent systems is the ability to have multiple entities that will interact with differential capacities to reach a solution to a proposed problem through cooperation, coordination, and negotiation [19]. In an example of one healthcare setting, GUARDIAN, a MAS works on a hierarchal basis in which each agent is split into perception/action, reasoning, and control agents [19]. There have also been proposals to have a hybrid MAS/E-health system to allow for greater efficiency in remote physician appointments, leading to more wholistic care [20]. One heavily researched MAS model for healthcare is the blackboard model seen below in Figure 2 [21]. 

Multi-agent systems exist to combine the efforts of these agents to provide a more wholistic spectrum of care, make efficient use of existing resources, and reduce costs to the system they are engaged in. MASs enhance complex problem-solving capabilities amongst healthcare system collaborators to provide effective care to various patient populations. By 2030, approximately 20% of the U.S. population will be older than age 65. Therefore, there is a growing need for a more cost-effective healthcare decision-making platform [22,23]. Multi-agent systems provide solutions for multiple healthcare sectors, such as organ transplantation and palliative care, due to the number of stakeholders required to make a shared medical decision [24,25]. Different stakeholders in healthcare include, but are not limited to, patients, doctors, medical institutions, governments, and medical insurance agencies [26]. MASs address a significant barrier within healthcare systems: interoperability [27]. In healthcare, solving problems requires the coordination of numerous individuals with different skills and functions, and the required knowledge is spatially distributed in different places [24]. To provide the best treatment possible, it would be of great benefit for all agents and knowledge to be significantly coordinated. This coordination begets a level of interoperability that the current systems do not have. Interoperability implements a space for the coordination of actions and the exchange of information [10]. The application of MASs within healthcare can monitor and coordinate all stakeholders who perform different tasks based on distinct information in order to devise a comprehensive solution to a patient’s problem. This makes them a viable solution in the managing, coordinating, controlling, and modeling of many different healthcare problems [28]. It quickly becomes more of a requirement than an option, particularly in health information systems [27]. 

MAS interoperability promotes other important characteristics, such as proactivity and autonomy, that are applicable to healthcare services [24]. These services include locating medical centers with certain specifications (i.e., palliative care in Houston, TX), accessing and/or updating medical records, and making an appointment to be evaluated by a specific physician [24]. Suppose a MAS is aware a patient has a specific medical condition and is traveling. In that case, it can proactively search for information regarding medical centers in town that can address their medical condition in case of an emergency [24]. The proactive behavior of agents allows actions that are not directly requested to be performed by rapidly retrieving, analyzing, and selecting required information for healthcare providers while also simultaneously integrating access to huge online information services [28]. Regarding autonomy, each stakeholder operates based on its knowledge and information retrieved from its domain [24]. However, MASs permit independent, autonomous entities to communicate and coordinate succinctly for pertinent determinations about patient care and thrive in dynamic and unpredictable environments [24,29]. 

There are multiple advantages to MAS implementation in healthcare. MASs perform well at addressing the goals of complex healthcare systems, including correct health data management, providing users with appropriate information to enhance the integrity and quality of healthcare, timely and accurate access to information, and minimizing medical errors [29]. They can be useful in monitoring patients through continuous assessment of symptoms and signs of disease, checking compliance with self-management programs, allowing improvement of treatment and patient outcomes, resource use, and effectively decreasing the cost of healthcare [30]. For example, the importance of follow-ups in patients with chronic heart failure (CHF) resides in reducing common causes of re-admission and deterioration of health status, which ultimately, if not mitigated, will impose physical and spiritual costs on both patients and society [30]. The continuous monitoring and appropriate application of proactive, effective, and affordable resources can decrease the burden on both patients and society. MASs can also address barriers to access to healthcare by minimizing unnecessary physical medical appointments through the provision of real-time data on patient statuses to healthcare providers. The obvious streamlining that this system would provide warrants study of the downstream effects it could potentiate amongst healthcare worker satisfaction and burnout rates.

## 4. Clinical Considerations

### 4.1. Trajectories

It is important to understand the clinical considerations when approaching the topic of palliative care. In general, it first becomes essential to know when palliative care is clinically appropriate. A 2005 clinical review from the United Kingdom looked through a database of papers, conducted a Medline search, and looked back at their previous palliative care studies to examine the idea that diseases have different illness trajectories [31]. Based on the trajectory and time course, different approaches to palliative care are required. The first clinical scenario involves a rapidly deteriorating illness such as cancer, which leads to rapid death within months or years. An illness can also be categorized as a long-term illness with periodic sudden declines. This can include disease processes such as congestive heart failure [32]. This second trajectory varies between individuals and always follows a steady decline with intermittent disease exacerbations. Lastly, the third trajectory consists of a slow decline. An example of this trajectory would include Alzheimer’s disease. Many clinicians still look to palliative care in the traditional sense only for those with rapidly progressive illnesses. However, palliative care must be considered for malignant and nonmalignant illness, alongside the appropriate curative treatment as seen in Murray et al study on heart failure vs lung cancer [33,34]. This can empower and potentially lead to better health outcomes for patients. Additionally, it can significantly improve quality of life [15,16,17,18]. In a systematic review of integrated palliative care in Europe, Siouta et al. found that patients offered palliative care were more likely to experience better symptom control, less caregiver burden, improvement in continuity and coordination of care, fewer admissions, cost effectiveness, and patients dying in their preferred place [35]. While the benefit of palliative care has been repeatedly demonstrated, the cost of the service and the need for greater amounts of palliative care professionals if instituted as a national healthcare standard is still problematic. 

### 4.2. Comparative Studies and Efficacy

A cross-sectional comparative cohort study examined the need for palliative care among patients with heart failure and cancer. Fifty heart failure patients and fifty cancer patients were followed to assess their overall need for palliative services. The study concluded that the two groups had comparable levels of palliative care needs. The study found that heart failure patients should not be excluded from specialist palliative care services. However, the primary team managing the patient must establish a ‘transition point’ when the patient needs palliative care [36]. A quality improvement report published in the Journal of Pain and Symptom Management examined the efficacy of Early Palliative Care Intervention (EPCI) in end-stage liver disease patients awaiting a liver transplant. The study concluded that EPCI led to an improvement in patient symptoms and depression. It also pointed out that specialized palliative care may be an underused service that may lead to objective improvements for patients [37]. Another study published in the New England Journal of Medicine found that patients offered early palliative care intervention when diagnosed with non-small-cell lung cancer had improved mood, greater resuscitation documentation, and less aggressive end-of-life care [38]. In a review of palliative care and heart transplant patients, Muhandiramge et al. reviewed 78 articles discussing clinical trials in heart failure patients on palliative care, and found that it significantly improved quality of life and survival rate [39]. In a study of 23,154 patients with advanced lung cancer, those offered palliative care 31–364 days after diagnosis were found to have increased life expectancy. Those on palliative care were also found to have reduced risk of death in acute care settings [40].

### 4.3. Safety

Patient safety can be another major concern in palliative care. A mixed-methods study looked at the national database of the National Health Service in the United Kingdom. It explored reports that looked at serious safety incidents in patients receiving palliative care. This study was conducted over 12 years from 2002 to 2014. The adverse events were classified into the following categories: pressure ulcer development or worsening, medication errors for end-of-life drugs, falls, healthcare-associated infections, and disturbed dying. The study concluded that safety incidents often resulted from a lack of coordination and systemic errors [41]. The lack of coordination that arises in these instances may be mitigated significantly via the use of a MAS. With a well-developed MAS, one could have care plans specific for said patient, leading to faster recovery and the avoidance of negligent conditions such as pressure ulcers. 

### 4.4. Multi-Agent System

A MAS may be useful in tackling the upcoming demand for palliative care and may also help decrease safety incidents. PalliaSys is a Spanish research project that has implemented MASs in the field of palliative care. Artificial intelligence and intelligent agents have allowed for continuous monitoring of patients and a warning to physicians and staff before a potential issue may arise. Additionally, the use of such systems can significantly improve the coordination of care for each unique individual patient. However, more research needs to be completed to assess the effectiveness of MASs in the palliative care setting [42]. Ultimately, the shortcoming of MASs lies in the lack of real-world implementation of healthcare variants despite promising studies and successful implementation within other industries such as logistics, transportation, utility management, and defense [43]. Clinical efficacy, safety, and comparative studies are highlighted in Table 1 and Table 2.

## 5. Conclusions

The aging population within the US is growing and will continue to cause greater strain on the healthcare system. If instituted as a healthcare standard for patients with chronic illness, palliative care services powered by MASs could satisfy the requirements of an ever-growing chronic illness-stricken population, while also providing high-quality healthcare. The use of MASs may prove to be a necessary paradigm shift in healthcare as the chronic disease patient population continues to expand. While many efforts have been made to prevent chronic disease before it begins, it is imperative that greater care be offered to those already suffering from it. Given the success of several studies that have shown the advantageous nature of MASs as a healthcare standard, this system is worthy of further investigation and potentially implementation as a staple healthcare system that may vastly improve our health as a nation. 

## Figures and Tables

**Table 1:** Clinical Efficacy and Safety.

Author (Year)	Groups Studied/Intervention	Results/Findings	Conclusions
Study 1: Baumann AJ et al. The benefit of Early Palliative Care Intervention in End-Stage Liver Disease Patients Awaiting Liver Transplantation [37]	A 2015 improvement report looked at patients with end-stage liver disease (ESLD). The patients were provided an early palliative care intervention and subsequently assessed to see if clinical improvement occurred in addition to positive changes in mood.	Pre-palliative care: 23 of 30 (76.6%) of patients reported moderate-to-severe symptoms of pruritis, well-being, anxiety, appetite, and fatigue. Pre-palliative care: 13 of 30 patients reported depressive symptoms. Post-palliative care: 50% of the moderate-to-severe symptoms significantly improved, with fatigue and well-being having less statistically significant reduction. Post-palliative depressive symptoms were reduced by 27.8%.	Study 1 provides objective data showing symptom improvement in those provided the palliative care intervention. This efficacy is significant for providers to keep in mind for all trajectories of illness. Knowing the efficacy of this palliative care intervention, the healthcare system must deal with the best method to cope with the increase in demand for services if it becomes widely used.
Study 2: Yardley I, et al. Patient safety in palliative care: A mixed-methods study of reports to a national database of serious incidents [41]	A 2018 study looked at the national database of the National Health Service in England to find reports of serious incidents requiring investigation. These reports were targeted at patients receiving palliative care.	475 reports identified. Reports classified as follows: 266 reports of pressure ulcers, 91 reports of medication errors, 18 of disturbed dying, 8 of transfer incidents, 6 of suicides, 5 unspecified.	The study concluded that these incidents could mostly be attributed to lack of coordination, staff and providers without proper palliative care experience, and under-resourcing.
Study 3: Sullivan D, et al. Association of Early Palliative Care Use With Survival and Place of Death Among Patients With Advanced Lung Cancer Receiving Care in the Veterans Health Administration [40]	A 2019 study looked at 23,154 patients with Stage IIIb or Stage IV lung cancer from the Veterans Affairs healthcare system. The study assessed enhanced survivability in early palliative care; 57% received palliative care.	Palliative care, after diagnosis, from 0–30 days = decreased survivability. Palliative care from 31–365 days, after diagnosis, =greater survivability. Palliative care received after 365 days = no significance.	Palliative care is associated with greater survivability and reduced risk of death in acute care settings. Palliative care should be considered a complementary approach to disease-modifying therapy in patients with advanced lung cancer.

**Table 2:** Comparative Studies.

Author (Year)	Groups Studied and Intervention	Results and Findings	Conclusions
Study 1: O’Leary N et al. A comparative study of the palliative care needs of heart failure and cancer patients [36]	A 2009 cross-sectional comparative cohort study looked to assess whether the palliative care needs of those with heart failure were similar to the needs of those with cancer. The study used both quantitative and qualitative measures in the study.	The two groups reported comparable levels of overall need for palliative care. Differences in specific needs did exist between the two groups. For example, the group with heart failure had a completely different symptom burden than the group with cancer.	A tailored approach must be considered with palliative care. It can benefit patients of all illness trajectories. However, each illness requires different needs to increase the comfort of the patient.
Study 2: Siouta N. et al., Integrated palliative care in Europe: a qualitative systematic literature review of empirically-tested models in cancer and chronic disease [35]	Fourteen studies including 7 for chronic disease, 4 for oncology, 2 for chronic disease and cancer, and 2 for end-of-life pathways. Evaluation of integrated palliative care in disease treatment throughout Europe.	Better symptom control, less caregiver burden, improvement in continuity and coordination of care, fewer admissions, lower costs, and patients passing in their preferred location.	A generic framework for PC in cancer and chronic disease is needed—one that includes aspects of treatment, consulting, and training.

## Data Availability

Data sharing is not applicable to this article as no datasets were generated or analyzed during the current study.
